# Platelets as messengers of early-stage cancer

**DOI:** 10.1007/s10555-021-09956-4

**Published:** 2021-02-26

**Authors:** Siamack Sabrkhany, Marijke J. E. Kuijpers, Mirjam G. A. oude Egbrink, Arjan W. Griffioen

**Affiliations:** 1 Department of Physiology, Cardiovascular Research Institute Maastricht, Maastricht University, Maastricht, The Netherlands; 2 Department of Biochemistry, Cardiovascular Research Institute Maastricht, Maastricht University, Maastricht, The Netherlands; 3 Angiogenesis Laboratory, Cancer Center Amsterdam, Department of Medical Oncology, VU University Medical Center, Amsterdam UMC, Amsterdam, The Netherlands

**Keywords:** Platelets, Biomarker, Early-stage, Cancer

## Abstract

Platelets have an important role in tumor angiogenesis, growth, and metastasis. The reciprocal interaction between cancer and platelets results in changes of several platelet characteristics. It is becoming clear that analysis of these platelet features could offer a new strategy in the search for biomarkers of cancer. Here, we review the human studies in which platelet characteristics (e.g., count, volume, protein, and mRNA content) are investigated in early-stage cancer. The main focus of this paper is to evaluate which platelet features are suitable for the development of a blood test that could detect cancer in its early stages.

## Introduction

Cancer is one of the foremost causes of death worldwide [1]. Over the past decades, researchers have been focused on the discovery of new approaches for improved therapy of cancer, ultimately aiming for cure. It is known that an earlier detection of cancer will profoundly improve the success of patient treatment and enhance overall survival [2–4]. However, early detection of cancer is notoriously difficult and efficient blood-based biomarkers are hardly available. Up to now, many blood-based sources of biomarkers, such as plasma, serum and circulating RNA/DNA, tumor cells, or exosomes/microparticles, have been exploited in the search for the ideal biomarker allowing detection of cancer at its earliest stages [5]. Remarkably, platelets have long been neglected in blood biomarker research [6], in spite of the growing evidence that platelets are important in the development and progression of cancer [7–9]. Platelets have been shown to possess an important biological role at several stages of malignant disease, such as angiogenesis [10], cell proliferation [11], cell invasiveness, and metastasis [12, 13]. In addition, there are indications that inhibition of platelet function has an inhibitory effect on tumor growth and that this increases overall survival of patients [14–17].

The interaction between platelets and cancer is evidently reciprocal [18]. Platelets have a stimulatory effect on cancer progression [7, 8], while at the same time, the presence of a malignant disease affects multiple platelet characteristics and functions. For example, malignant tumors have been shown to increase platelet numbers and hijack platelet functions in order to fuel cancer progression [19]. Moreover, there are several promising studies showing that platelet features from patients with cancer are already altered in early stages of malignant disease [20–24]. Hence, the use of platelet characteristics is expected to provide an innovative strategy in the search for biomarkers of early-stage cancer [9]. In the current paper, we will briefly review the mechanisms by which platelets stimulate cancer progression and present an overview of literature describing the effect of cancer presence on platelet activation, count, volume, mRNA/protein content, and function, as well as whether these features could be used as biomarkers of early-stage cancer.

## Platelets promote angiogenesis, tumor growth, and metastasis

**Fig. 1:**
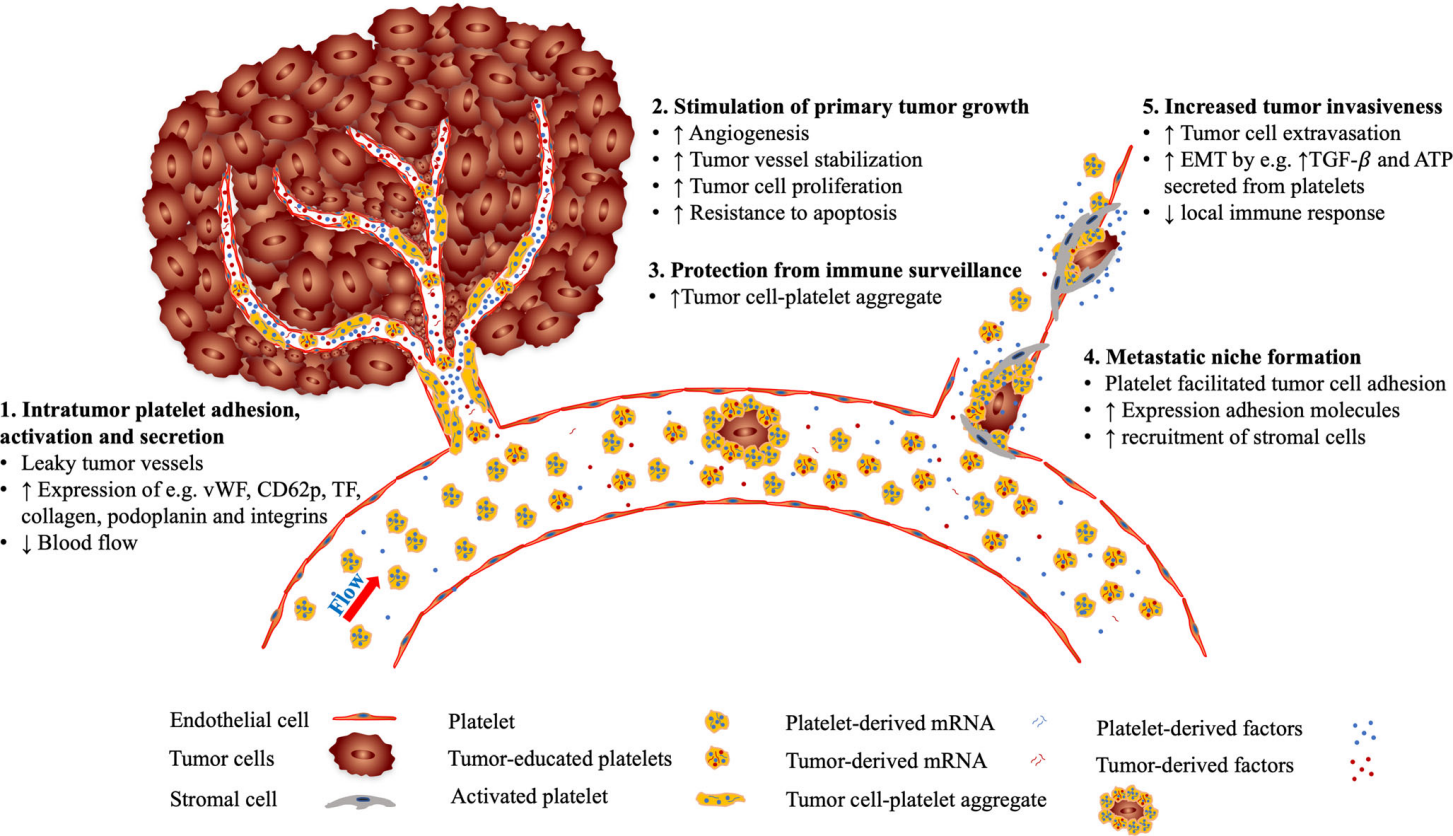
Platelets promote tumor angiogenesis cancer growth and metastasis. (1) The prothrombotic tumor microenvironment induces platelet adhesion, activation, and secretion in the (angiogenic) blood vessels within the tumor. (2) Platelet secretome and microparticles induce tumor angiogenesis, vessel stabilization, cancer cell proliferation, and resistance to apoptosis. At the same time, platelets sequester proteins and mRNA from the tumor (and become so-called tumor-educated platelets). (3) Tumor cell-platelet aggregate formation shields circulating tumor cells from the immune system. (4) Platelets support metastatic niche formation by inducing the expression of adhesion molecules and recruitment of stromal cells at potential metastatic sites. (5) Platelet secretome increases tumor cell invasiveness by inducing epithelial-mesenchymal transition (EMT) and inhibiting local immune response

Cancerous tumors are also viewed as wounds that never heal [25], because they induce local and systemic coagulation and platelet adhesion, activation, aggregation, and secretion [26–28]. During the past decades, it has become increasingly clear that tumors can use platelets to stimulate angiogenesis and induce cancer cell proliferation and metastasis (Fig. 1) [7, 10, 29].

The composition of the tumor vasculature is different from the normal vasculature [30, 31]. Blood vessels in a tumor are characterized as immature and leaky, due to the exposure to the tumor microenvironmental milieu. Hence, the tumor endothelium can be discontinuous, providing prothrombotic conditions via the expression of thrombotic proteins, e.g., tissue factor, collagen, von Willebrand factor, podoplanin, and integrins [32–36]. Eventually, these factors, combined with the low blood flow, create a thrombophilic tumor microenvironment [25, 37]. This prothrombotic state initiates platelet adhesion and activation, followed by secretion of the rich granule content of platelets within the tumor [10].

Platelet granules contain an enormous amount of bioactive growth factors, e.g., chemokines, cytokines, and matrix metalloproteinases (MMPs). Upon secretion, these molecules can support tumor growth and progression, mainly via, but not limited to, induction of tumor angiogenesis [10, 38]. This rate-limiting step in cancer growth is intricately regulated by a plethora of growth factors and the involvement of tumor cells and different tumor stroma cells (e.g., endothelial cells, pericytes, smooth muscle cells, fibroblasts, platelets, and a variety of mature and immature immune cells) [39]. The role of platelets in this process is slowly becoming elucidated [7, 10, 40–43]. By secreting their bioactive molecules within the tumor microenvironment, platelets not only play an important role in the regulation of tumor angiogenesis but also in vascular stabilization and integrity [41–45], as well as resistance to therapy [46, 47].

It has been described that the secretome and microparticles released from activated platelets increase proliferation of several cancer cell lines (e.g., ovarian and hepatocellular carcinoma cancer cells) [11, 48]. In addition, platelets are able to increase resistance to mitochondrial apoptosis in cancer cells [41, 42, 49]. At the same time, platelets support tumor cell survival and transport in the circulation once these cells detach from the primary tumor site. To this end, platelets are able to attach to circulating tumor cells via several adhesion receptors, which results in the formation of tumor cell-platelet aggregates [50]. Exploiting this mechanism, circulating tumor cells are able to protect themselves from immune surveillance [50, 51]. Platelets aggregated to the tumor cell surface also support adhesion to the endothelium of potential metastatic sites [52]. Platelet-vessel wall interaction induces the expression of multiple adhesion molecules on the luminal side of the vessels which increases recruitment of stromal cells (e.g., monocytes) [51, 53]. In addition, CD97 expressed on tumor cells results in platelet activation and secretion of platelet-derived mediators, such as ATP, lysophosphatidic acid, and TGF-β, that disrupt endothelial junctions and thereby increase tumor cell invasiveness and extravasation into healthy tissue [12, 54]. Furthermore, TGF-β secreted from platelets and direct platelet-tumor cell contact activates the TGF-β/Smad- and the NF-κB pathways in malignant cells [12]. This stimulates enhanced tumor cell aggressiveness by epithelial-mesenchymal transition (EMT) [12, 13, 52]. Platelets can induce cancer cells to upregulate mesenchymal markers such as SNAIL, vimentin, fibronectin, and MMP-9, and stimulate downregulation of E-cadherin, which is a fundamental step in EMT [12, 55]. At the same time, TGF-β downregulates the local immune response, for example, by inhibiting the expression of NKG2D, the major receptor of several MHC class I homologs, on natural killer cells [13, 56].

## Platelet inhibition reduces cancer progression

As tumors use (activated) platelets to boost tumor angiogenesis, tumor growth, and metastasis (Fig. 1), it has been suggested that targeting platelets may result in inhibition of cancer progression. Indeed, reduction of platelet count in tumor-bearing mice reduced tumor angiogenesis, tumor growth, and metastasis [41, 42, 57–59]. Besides their stimulatory effect on tumor growth, platelets are critical at stabilizing the tumor vasculature and preventing intratumoral hemorrhage [41, 43]. In addition, multiple *in vivo* studies have shown that treatment with aspirin or clopidogrel, both inhibitors of platelet aggregation, reduces tumor angiogenesis and tumor growth, as well as cancer progression in tumor-bearing mice [17, 44, 60–63]. Furthermore, large epidemiological studies suggested that daily intake of aspirin at low anti-platelet doses reduces the risk of development of several types of cancer [14, 16]. Also, a prospective cohort study revealed that daily aspirin intake after the diagnosis of colorectal cancer decreased cancer-specific death and overall mortality [64]. Although the exact mechanism underlying this effect of aspirin is still poorly understood, several recent studies suggest that inhibition of platelets by aspirin reduced their ability to induce cancer cell proliferation through modulation of the c-MYC oncoprotein and inhibition of platelet-derived COX-1/thromboxane A2 [17, 26]. However, the effect of aspirin on tumor growth could also be due to its inhibitory effect on COX-2 expressed on tumor cells [65, 66]. Large randomized placebo-controlled prospective human studies investigating the effect of platelet inhibition on overall survival of patients with cancer are ongoing, and may provide further clinical evidence of the protective properties of anti-platelet agents against cancer [67].

Over the past few years, there are also an increasing number of studies indicating that certain anticancer therapies, specifically tyrosine kinase inhibitors (TKIs), reduce platelet count, as well as platelet activation [68–70]. This class of drugs is widely used for the clinical management of a variety of cancer types, mostly in combination with more conventional treatment strategies. It is therefore assumed that next to the direct effect of these drugs on tumor cells, they may lend part of their activity indirectly via the inhibition of platelet function.

## Tumor cells influence platelet characteristics

Activated platelets stimulate cancer progression at different stages, making platelets an attractive target in the battle against cancer. At the same time, the presence of a tumor has a major influence on platelet characteristics, possibly through effects at the level of the megakaryocytes. These characteristics include platelet count, volume, protein and mRNA content, and activation state; responses at the level of these features make platelets an interesting new target for blood biomarker research for the detection of early-stage cancer [9].

### Platelet count

**Fig. 2:**
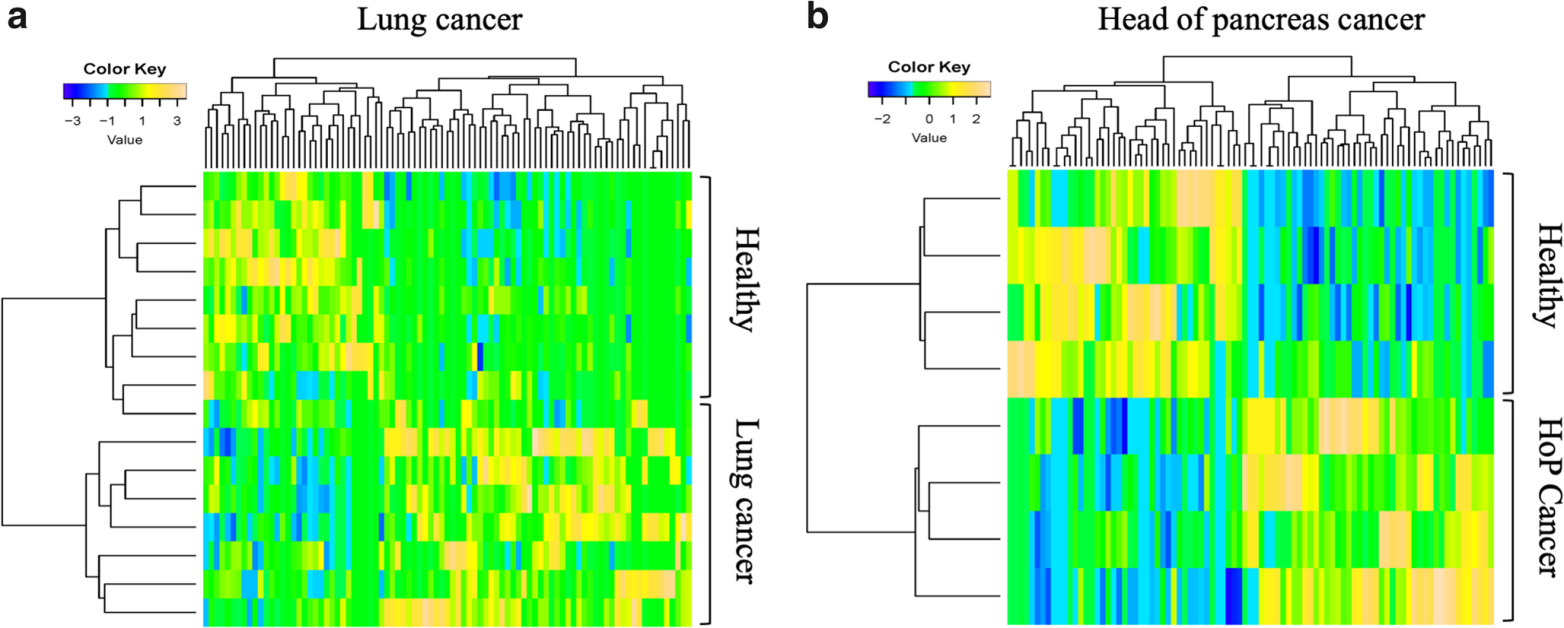
Heat map and supervised cluster analysis of protein expression data from platelets of early-stage lung or head of pancreas cancer (HoP cancer) patients compared to healthy sex- and age-matched controls. Cancer type-specific supervised cluster analysis clearly separates the platelet proteome of patients with early-stage lung cancer (**a**) or head of pancreas cancer (**b**) from healthy controls. Reprinted from [10] with permission

The association between platelet count and malignant disease is well recognized. In the presence of occult cancer, platelet production can be heavily increased in response to various tumor-derived and systemic factors (Fig. 2) [7, 19]. Platelets are produced in the bone marrow through the formation of proplatelets by terminally differentiated megakaryocytes [36]. *In vivo* studies have demonstrated that tumor-bearing mice have increased serum levels of megakaryocyte-stimulating factors, such as IL-6, M-CSF, and SDF-1α [71]. Also in patients, plasma levels of G-CSF, GM-CSF, and IL-6 were found to be elevated, resulting in enhanced platelet production and paraneoplastic thrombocytosis [72]. Pucci et al. described platelet factor-4 (PF-4) as a cancer-enhancing endocrine signal-stimulating bone marrow megakaryopoiesis, which is associated with a decreased survival of lung cancer patients [73]. However, the chicken-or-the-egg question is still unanswered: either an increase in thrombopoietic factors leads to an increase in platelet count with subsequent stimulation of tumor growth, or an already aggressive tumor secretes thrombopoietic factors, which results in an increase in platelet count. Nonetheless, increased levels of thrombopoietic factors and elevated numbers of platelets are often observed in patients with cancer.

We and others have demonstrated a clear correlation between platelet count and the presence of cancer [19, 23, 74]. Also, there is accumulating evidence that thrombocytosis (i.e., a platelet count above 400 × 10^9^ platelets/L) is an independent predictor of poor prognosis in various types of cancer [19]. However, the value of platelet count as a biomarker of early-stage cancer is still unclear. A large clinical study with approximately 140,000 patients showed that almost 40% of patients with idiopathic thrombocytosis (i.e., without inflammatory disease or iron deficiency) exhibit some form of occult malignancy [75]. In addition, a recent systematic review of studies performed in a primary care setting suggests that thrombocytosis is a marker of increased risk of cancer presence in adults older than 40 years [76]. In this study, the incidence of cancer in the year following the assessment of thrombocytosis was 11.6% in males and 6.2% in females, as compared to 4.2 and 2.2% in males and females with a normal platelet count, respectively. Interestingly, in this study, thrombocytosis was the strongest predictor of lung and colorectal cancer. While the studies above suggest that thrombocytosis could be a risk factor of undiagnosed cancer in primary care setting, it is not specified whether these cancer diagnoses concerned early- or late-stage cancer. Overall, platelet count may be a good predictor of poor prognosis in patients with cancer; however, platelet count as a single measurement is probably not sufficient for detection of early-stage cancer.

### Platelet volume

Platelet volume is established during platelet formation, where preplatelets are transformed to proplatelets [77]. Platelet volume (normal range: 9.4–12.3 fL) is genetically determined and is fairly stable over the lifetime of healthy individuals [78], but it can vary during a wide range of diseases. Changes in mean platelet volume (MPV) have been demonstrated in cardiovascular disease, cerebrovascular disease, peripheral artery disease, Crohn’s disease, and colitis [79–83]. In addition, from studies by us and others, it is becoming increasingly clear that MPV is also affected during malignant diseases [23, 84–87]. Electron microscopy has revealed that platelets of patients with ovarian tumors have more mitochondria and significantly smaller microtubules compared to platelets from healthy individuals [88]. We demonstrated that MPV is elevated in patients with lung or head of pancreas cancer [23]. Furthermore, a recent meta-analysis confirmed that the MPV of treatment-naïve patients with malignant tumors was significantly higher compared to the MPV of healthy individuals, which generally seemed to normalize after treatment [84].

Over the past years, accumulating evidence suggests that the platelet population within one individual is heterogenous in platelet structure and activation properties [78, 89]. This heterogeneity is most likely the result of differences during thrombopoiesis (e.g., by dissimilar megakaryocyte proplatelet formation), imbalanced platelet priming, environmental conditions and platelet ageing [90]. Therefore, changes in MPV could be a reflection of proinflammatory and/or prothrombotic conditions where thrombopoiesis is affected by inflammatory cells and bioactive molecules such as IL-6 and C-reactive protein [91]. It is suggested that the largest platelets in an individual are associated with increased platelet aggregation, beta-thromboglobulin secretion, thromboxane synthesis, and metabolic and enzymatic activity [92, 93]. Consequently, larger platelets can be assumed to be more reactive, as compared to smaller platelets as suggested in patients with coronary artery disease [92]. This increased reactivity is, among others, due to an increase in copy numbers of integrin αIIbβ3 and glycoprotein (GP) Ibα on larger platelets [94, 95]. This is in agreement with other studies, where an increase in platelet activation is demonstrated in patients with cancer [23, 69, 96]. In addition, several retrospective and prospective studies suggest that larger platelets are more reactive and that patients with large platelets are at higher risk for thrombotic events [97–99]. It is important to realize, however, that platelet volume is not the only factor determining platelet reactivity to stimuli [78, 90].

Thus far, the impact of MPV in patients with malignant tumors is not fully understood.

There are several studies, which reveal that high MPV is a predictor of poor prognosis in various types of cancer [85, 100–102]. However, the role of MPV as an early messenger of cancer presence seems to be limited, as it is mostly affected in later stages of the disease [86, 87, 100, 103]. In addition, while in most studies the differences in MPV between patients with advanced cancer and controls are significant, the changes in platelet volume between patients and controls are very small. For example, MPV has been shown to be significantly higher in patients with gastric or endometrium cancer compared to healthy controls [86, 104]. However, MPV was 8.31 fL (range 7.53–9.09) or 7.8 fL (range 6.2–11.3) in patients with gastric or endometrium cancer, respectively, whereas in healthy controls, MPV was 7.85 fL (range 7.4–8.3) and 7.2 fL (range 1.6–14.9), respectively. These small differences can be easily masked by a wide range of variables that can impact MPV. Therefore, MPV as a single measurement does not seem to be a good marker to distinguish patients with early-stage cancer from healthy individuals.

### Platelet protein content

Platelets transport a vast amount of bioactive proteins in their granules, including growth factors, chemokines, and proteases, which they can secrete upon activation [10]. *In vivo* studies with mice bearing human tumors have demonstrated that platelets are able to sequester proteins that are secreted from tumors [71]. This resulted in higher concentrations of tumor-derived factors (such as TGF-β, MCP-1, RANK, and TIMP-1) inside platelets due to the presence of cancer [71]. In addition, platelets of tumor-bearing mice were observed to contain increased levels of thrombospondin-1 (TSP-1), which is highly correlated to tumor progression. After resection of the tumor, these levels decreased to baseline [105]. Upregulation of proteins, such as TSP-1 and platelet factor 4 (PF4), inside platelets could even be used to detect clinically undetectable tumors (< 1 mm^3^) in mice [105, 106].

Multiple studies in humans have also demonstrated changes in platelet protein content in patients with cancer (Table 1). However, part of these results are derived from indirect measurements where the platelet content was not measured but calculated ($\mathrm{Platelet}\ \mathrm{content}=\frac{\mathrm{Serum}\ \mathrm{concentration}-\mathrm{plasma}\ \mathrm{concentration}}{\mathrm{Platelet}\ \mathrm{count}\ \mathrm{in}\ \mathrm{whole}\ \mathrm{blood}}$) from serum and plasma concentrations. Nevertheless, there are also studies where bioactive factors were directly measured in isolated platelets from patients with cancer and compared to platelets of healthy individuals [20, 23, 107]. Peterson et al. demonstrated elevated concentrations of VEGF, PF4, and PDGF in platelets of patients with colorectal cancer [20]. In addition, they showed that these changes in platelets provided a statistically significant discrimination between patients and age-matched healthy controls [20]. While approximately half of the patients included in this study had early-stage (I–II) colorectal cancer, the small sample size prevented detection of a correlation between cancer stage and platelet content. In our study of 2017 [23], we demonstrated a significant increase in concentrations of VEGF and PDGF in platelets of patients with early-stage (I–II) lung cancer compared to platelets of a healthy sex- and age-matched control group. In platelets of patients with late-stage (III–IV) lung cancer, we observed a decrease in PF4, CTAPIII, and TSP-1 as compared to the control group, which was similar to a previous study performed in patients with advanced cancer [107]. In patients with head of pancreas cancer only, VEGF was elevated in platelets as compared to controls [23]. Thus, changes in platelet protein content are consistently observed in patients with cancer. Interestingly, the platelet changes appear to be tumor-type-dependent as demonstrated by multiple other studies (Table 1). The combination of changes in multiple platelet characteristics allowed cancer type-specific discrimination between early-stage cancer patients and healthy controls. For this purpose, we used the data from our study to develop a multivariable diagnostic model for both lung and head of pancreas cancer, including platelet count, volume, protein content, and activation status [23]. This model appeared to be able to distinguish patients with early-stage lung or head of pancreas cancer from healthy sex- and age-matched controls [23]. Overall, the above studies suggest that a combination of platelet measurements could be used to distinguish patients with early- or late-stage cancer from healthy individuals.

**Table 1:** Expression of proteins in platelets of patients with different cancer types compared to control

Protein	Early-stage (I–II)	Advanced-stage (III–IV)	Stage not specified (I–IV)
Ang-1			PC~[117], BC↑[117]
bFGF			HCC↑[118], CRC~[20]
Clusterin	CRC↓[119]	CRC↓[119]	
Cofilin-1	CRC↑[119]	CRC↑[119]	
CTAPIII	LC~[23], HoP~[23]	LC↓[23], HoP~[23]	
CXCL12		VC↓[107]	
Endostatin			HCC↑[118], CRC~[20], GBM~ [120]
GSH-s	CRC↑ [119]		
HGF			HCC↑[118]
PDGF	LC↑[23], HoP~ [23]	LC~[23], HoP~[23]	CRC↑[20, 121], BC↑[122], HCC~[118]
PF4	LC~[23], HoP~[23]	BC↑[123], LC↓[23], VC↓[107], HoP~[23]	CRC↑[20], BC↑[122], HCC~ [118]
TGF-β			BC↑[122], BC~[124]
TSP-1	LC~[23], HoP~[23]	LC↓[23], VC↓[107], HoP~ [23]	BC~[122, 124], CRC~[20], HCC~[118]
VEGF	LC↑[23], HoP↑[23], BC↑[125]	LC↑[23, 126], HoP↑[23], BC↑[123, 125]	CRC↑[20, 121], GBM↑[120], HCC↑[118], PC↑[117], BC↑[122, 124, 127]

Expression (increased(↑), decreased(↓), not changed(~)) of proteins in platelets of patients with lung (LC), colorectal (CRC), breast (BC), glioblastoma (GBM), head of pancreas (HoP), hepatocellular (HCC), prostate (PC) cancer or mix of various cancers (VC) compared to a (healthy) control group. Empty spaces indicate a lack of suitable research

*Ang-1* angiopoietin-1, *bFGF* basic fibroblast growth factor, *CTAPIII* connective tissue activating peptide-III, *GSH-S* glutathione synthetase, *HGF* hepatocyte growth factor, *PDGF* platelet-derived growth factor, *PF4* platelet factor-4, *TGF-β* transforming growth factor beta, *TSP-1* trombospondin-1, *VEGF* vascular endothelial growth factor

The changes in platelet content are not limited to the abovementioned proteins. Several years ago, Klement and colleagues demonstrated a substantial change in the platelet proteome of tumor-bearing mice [108]. In 2018, we have performed the first study in humans, in which 139 differentially expressed proteins were found in platelets of patients with early-stage lung or head of pancreas cancer as compared to healthy individuals of comparable age and gender [22]. Furthermore, surgical removal of the malignant tumor resulted in normalization of the platelet proteome [22]. Interestingly, we found that molecular content changes in head of pancreas cancer were more pronounced and showed a different profile as compared to the lung cancer platelet proteome, which supports the notion of tumor-type dependency of platelet changes (Fig. 2). Multiple potential platelet-derived biomarkers of early-stage cancer were identified by the comparison of the platelet proteome in cancer patients with that of sex- and age-matched healthy controls [22].

Several mechanisms underlie the changes in platelet proteome of patients with malignant disease (Fig. 1). Proteins present in platelets are either synthesized by megakaryocytes that produce the platelets, or are absorbed by megakaryocytes and/or platelets themselves from the blood [105, 108, 109]. The concept of tumor-educated platelets (TEPs) assumes that platelets can directly take up bioactive factors from tumor cells, while residing temporarily in the tumor [21, 22]. As a consequence, the platelet proteome mirrors the tumor-based changes and the concentration of potential tumor reporter proteins can be considerably higher inside platelets than in plasma. This enables more accurate measurement, as compared to quantification in plasma, making platelets an attractive source of biomarker discovery [20, 23, 108]. Several studies even suggest that in the presence of a growing tumor, changes inside platelets precede alterations in plasma [20, 105, 106, 108]. This implies that measurements of potential biomarkers inside platelets could be more sensitive for detection of early-stage cancer than plasma measurements.

### Platelet mRNA content

The platelet mRNA profile is currently emerging as a new potential source in cancer biomarker research [21, 24, 110]. Platelets do not contain a nucleus and, therefore, the mRNA transcripts either originate from megakaryocytes during platelet synthesis [36, 111] or are absorbed from the blood during circulation and/or interaction with tumor or other cell types [112]. Several mechanisms have been described that determine the platelet mRNA profile. Nilsson et al. demonstrated tumor-derived mRNA transfer (mutant EGFRvIII) from cancer cells to blood platelets of healthy individuals *in vitro* [110]. In addition, they showed that platelets isolated from glioma or prostate cancer patients also contained the cancer-associated mRNA biomarkers EGFRvIII and PCA3. Most likely, platelets take up these mRNAs via microvesicle-dependent endocytosis [113], but a microvesicle-independent mechanism is also possible [114]. In addition, induction of platelet activation by external stimuli (e.g., collagen or bioactive molecules secreted by a tumor) stimulates maturation of resident pre-mRNAs in platelets and results in RNA splicing in platelets during activation [115].

The potential of using platelet mRNA as a diagnostic tool in patients with cancer was emphasized by a study of Best et al., who discovered changes in platelet mRNA profiles in cancer patients [21]. In this study, the platelet mRNA profile of patients with several types of cancer was compared to the platelet mRNA profile of healthy individuals. This study demonstrated that the platelet mRNA profile was affected in almost all cancer patients. A combination of changed mRNAs allowed good discrimination between cancer patients and healthy individuals with high sensitivity (96%) and specificity (92%). An important limitation of this study was the nature of the control group that was clearly younger and had a different gender distribution than most of the cancer groups. This is especially important as we and others have shown that changes in platelet characteristics are correlated with age and sex [23, 116]. In a follow-up study, Best et al. used a particle swarm optimization–enhanced algorithm to select an mRNA biomarker panel that could distinguish patients with NSCLC (non-small cell lung cancer) from healthy individuals [24]. A panel of 1000 genes resulted in accurate detection of early- and late-stage NSCLC when compared to an unmatched control group. Matched evaluation for age and smoking status (panel of 830 genes) also resulted in a statistically significant discrimination of patients with NSCLC from healthy controls. Unfortunately, the latter analysis was not subdivided in early- and late-stage cancer. Overall, platelet-derived mRNA seems to be a sensitive and promising new tool in the search for biomarkers of early-stage cancer. Further studies are still needed to validate these encouraging results.

## Conclusion

Early detection of cancer allows a significant improvement of overall survival of patients. During the past years, platelet characteristics are emerging as a promising source for biomarkers of cancer. This review summarizes and discusses studies in which platelet features (e.g., count, volume, protein, and mRNA profile) are explored in early-stage as well as late-stage cancer. Platelet count and volume appear to have a more prognostic value and their importance as biomarkers of early-stage cancer remains to be demonstrated. However, a combination of platelet count, volume, and selected platelet-derived proteins could be used to discriminate patients with early-stage cancer from healthy individuals. Most promising data are from patient studies illustrating vast changes in platelet proteome and mRNA content. These changes occur already in early stages of cancer and illustrate that the platelet content could be a rich source of potential biomarkers. In addition, these profiles could be exploited to provide information on the organ of origin, which could provide a lead for clinical follow-up diagnostics to confirm the tumor location.

Future large-scale studies are needed in general population or in people who have a genetic disposition to acquire certain types of cancer (e.g., patients with mutation in BRCA1/2 or Lynch syndrome). These individuals have a very high lifetime risk of developing cancer and are therefore frequently screened with invasive and/or imaging techniques to detect potential tumors. These patients are therefore an exceptional group to study, to investigate whether platelet changes precede clinically detectable tumors.
